# Usability and expert validation of a virtual reality system for post-mastectomy rehabilitation

**DOI:** 10.3389/fresc.2025.1718756

**Published:** 2026-01-12

**Authors:** Giulia Bongiorno, Ilaria Albi, Tommaso Coianiz, Helena Biancuzzi, Francesca Dal Mas, Daniele Vidi, Luca Miceli

**Affiliations:** 1 “Friuli Riabilitazione” rehabilitation center, Roveredo in Piano (PN), Italy; 2 University of Udine, Udine, Italy; 3 Department of Economics, Ca’ Foscari University of Venice, Venice, Italy; 4 Department of Management, Ca’ Foscari University of Venice, Venice, Italy; 5 Collegium Medicum SAN University of Lodz, Lodz, Poland; 6 Centro di Riferimento Oncologico IRCCS di Aviano (PN), Aviano, Italy

**Keywords:** breast cancer, physical activity, shoulder rehabilitation, tennis, virtual reality

## Abstract

**Introduction:**

Virtual reality (VR) is proposed as a support (and potential alternative) to traditional rehabilitation after breast cancer surgery, with expected effects on pain, anxiety, fatigue, and recovery of shoulder ROM (range of movement). The study aims to test a VR system that integrates body illusion, gamification, and distraction, evaluating its usability, user experience, and comfort for clinical use.

**Methods:**

The study was conducted on 31 healthy subjects (28 physiotherapy students, 3 physiotherapists). The software includes a shoulder ROM calibration phase and a training phase involving a VR tennis game, with adjustable parameters and automatic performance recording. SSQ, SUS, UEQ, CRS, and a question on the adequacy of playing speed were administered. A descriptive analysis (mean, standard deviation) and a qualitative analysis of the open-ended responses were conducted.

**Results:**

SSQ: very low average symptoms (minimal discomfort). SUS: good perceived usability (easy, integrated, safe). UEQ: Overall positive experience (clarity, modernity, pleasantness); concerns regarding predictability/slowness. CRS: High tolerability and low anxiety/harm; higher “attachment.” Regarding speed: 15 “Yes” votes to making it faster, 16 “No” votes (divided opinions).

**Discussion:**

The VR system is well tolerated, usable, and has a favorable user experience in healthy subjects, indicating promising clinical transferability for shoulder rehabilitation after breast surgery. Dynamics and predictability (adaptive speed/levels) remain to be optimized, and the software needs to be validated on patients and experienced physiotherapists in dedicated studies.

## 1 Introduction

There are just under 390,000 new diagnoses of malignant tumors (excluding skin cancers and melanoma) estimated in Italy in 2024: 214,000 new cases in men, while 175,000 new cases in women (1). Although conservative surgical treatment involves a more selective and less invasive technique than radical surgical treatment, where the entire breast is removed (2), the postoperative complications that may occur include: lymphedema, pain, reduced mobility of the upper limb and finally an alteration of the patient's quality of life (3). Virtual Reality represents an innovative approach to traditional rehabilitation by providing support to conventional rehabilitation methods in the management of pain, fatigue, anxiety and depression in certain pathologies such as Parkinson's, stroke, but also prostate and rectal cancer (4). Recent studies (5) demonstrate the use of Virtual Reality and its benefits in terms of psychological symptoms (anxiety and depression) and physical symptoms (pain, fatigue, and joint limitation) in women with breast cancer. According to the Global Cancer Observatory, in 2022, there were 2,296,840 new diagnoses of breast cancer globally, making it the second most common site of malignant tumor development after lung cancer (6). Postoperative complications make multidisciplinary patient care and management essential, providing constant personalized psychological, medical, and physiotherapy support (7). Among the measures implemented, physical activity plays a key role in minimizing the negative effects on the patient's quality of life and reducing postoperative complications, considering it equal to “adjuvant therapy” rather than a complement to oncological therapy (8). Furthermore, therapeutic training of the upper limb is also optimal in preventing the onset of lymphedema (8, 9). The heterogeneity of the pathology and its clinical manifestations makes multidisciplinary therapeutic management and the personalization of medical and physiotherapy treatments essential, through the development of rehabilitation programs and therapeutic exercises constantly monitored by the multidisciplinary team. Virtual Reality is defined as a digital environment generated by a computer that offers the user the possibility of interacting and immersing themselves in a non-real environment (10); furthermore, Virtual Reality is not only playful in nature but is increasingly gaining ground in the medical rehabilitation field, exploiting its characteristics of body illusion, distraction from pain and pain reduction (11–13). In the rehabilitation world, Virtual Reality is assuming an increasingly preponderant role, positioning itself as a supportive therapy, but also as a possible rehabilitation methodology to replace traditional musculoskeletal (14) and neurological rehabilitation (15, 16). In literature there are several studies regarding the use of Virtual Reality in oncological patients operated for breast cancer (17); these studies analyse the effectiveness of the virtual experience on motor limitations such as the reduction of joint ROM in the upper limb and muscle strength (5), but also the influence on the emotional and psychological sphere (18). An initial approach to the virtual world in the rehabilitative treatment of oncological patients consists in the use of commercial videogames; in fact, some authors (19, 20) propose the use of games for the “Xbox 360” console with the “Kinect camera”. The motivation and excitement in solving and carrying out the challenges and activities proposed by the virtual world makes the rehabilitative experience more engaging and captivating. A group of physiotherapists describe the applicability, appropriateness and acceptability of Virtual Reality by describing it as simple, clear and exciting to use (21). In addition, the user's involvement, given by immersion in the virtual experience, determines the distraction from his/her real clinical condition, allowing an increase in functional performance beyond expectations (22). Pain symptoms are more controlled and reduced, in fact the immersive and engaging nature of Virtual Reality lends itself as an effective means of distraction from the intensity of the painful symptom, increasing the pain tolerance threshold of oncological patients (5, 11). There are two characteristics of Virtual Reality that make it remarkably valid in the management of the symptoms commonly reported by women who have undergone breast cancer surgery: the distraction from the painful symptoms, with the consequent reduction in pain intensity (11) and the “gamification” process (12) that generates the transformation of the rehabilitation requests into a virtual game, determining an increase in motivation and the positive effects of the use of Virtual Reality. In addition to these characteristics, Virtual Reality presents further peculiarities that can be investigated in the context of the multidisciplinary rehabilitation management of women with breast cancer, in particular the mechanism of body illusion that defines the virtual world and leads the user to a fictitious representation of the self. A study (13) investigates how the manipulation of visual proprioceptive stimuli can alter the painful symptoms evoked by movement in patients who report neck pain; In fact, when the virtual proprioceptive visual feedback overestimates the real range of motion, the “pain-free” range is reduced compared to the usual range, contrary to what happens when the visual proprioceptive feedback underestimates the real range of motion, in which there is an increase in the pain-free range of motion. This latter result was also obtained by a further study (23): underestimating the joint range of motion allows for gains in joint excursion in real active neck rotation, particularly in individuals with an intense fear of movement. However, due to the particularly limited sample size, these results cannot be generalized: a further study (22) highlights that the fear of movement experienced by some subjects does not lead to an increase or decrease in neck joint range of motion compared to participants not fearful of movement. Finally, breast cancer survivors often experience long-term physical and psychological difficulties, including negative emotions and altered body image. Interoception, the awareness of internal bodily sensations, plays a key role in emotional regulation but can also contribute to anxiety and fear of cancer recurrence when overly focused on bodily symptoms. Virtual reality may help address these challenges by promoting embodiment, improving awareness of bodily sensations, and enhancing emotional wellbeing. Based on this framework, some authors propose a VR intervention aimed at increasing interoceptive capacities and improving body perception and emotional regulation in breast cancer survivors (24). Our working hypothesis is that a virtual reality system could offer advantages in several areas: gamification could lead to greater exercise compliance in future home-based patients; distraction could help avoid thinking about the entire psychological construct related to a breast cancer diagnosis; and the illusion of movement could reduce the fear of movement itself, an important psychological component in the rehabilitation process for these patients, as reported above. All of this, however, requires that the system offer a simple experience and usability that users appreciate. The following study aims to test a Virtual Reality system as a rehabilitation tool for shoulder dysfunction in women undergoing breast cancer surgery. This software integrates the features previously described: body illusion, “gamification,” and distraction. The software was tested on healthy subjects, physiotherapist students, and physiotherapists, whose experience was evaluated through the administration of four questionnaires that investigate the “User Experience”, the comfort of the software and its usability to make the system optimal and suitable for its future clinical use.

## 2 Methods

This study was conducted at the P.O. Gervasutta facility, home of the Physiotherapy Degree Program at the University of Udine, between June and September 2025. A total of 31 subjects were enrolled: 28 third-year physiotherapy students at the same university (average age 23.4 years, 19 males and 9 females) and three physiotherapists practicing at the same facility (average age 50 years). The overall average age of the sample was 26.7 years. Subjects suffering from motion sickness, ocular, neuromuscular, neurological, psychiatric, or significant hearing disorders were excluded from the study. Wearing glasses due to refractive errors was not considered an exclusion criterion. All subjects had a level of digital literacy that allowed them to use a smartphone and a virtual reality headset, and already had basic knowledge of post-surgical breast rehabilitation and related shoulder pathology. Virtual reality software dedicated to oncological rehabilitation has been developed at the Department of Mathematical, Computer, and Physical Sciences at the University of Udine. This software has been tested on healthy subjects, whose experience is evaluated by completing various questionnaires assessing user experience, software usability, and comfort. This aims to highlight any potential strengths and weaknesses to optimize the system for future clinical use. The software's operation is enabled by two phases:

Calibration phase: During this phase, the subject is asked to perform certain movements with the affected limb. The goal of this phase is to determine the position of the shoulder in virtual space and the active range of motion.
Training phase: During this phase, the subject plays tennis with the virtual pedagogical assistant. The racket is obtained by grasping the Meta Controller, and the surrounding scenario is represented by a red singles tennis court with a net.

**Figure 1:**
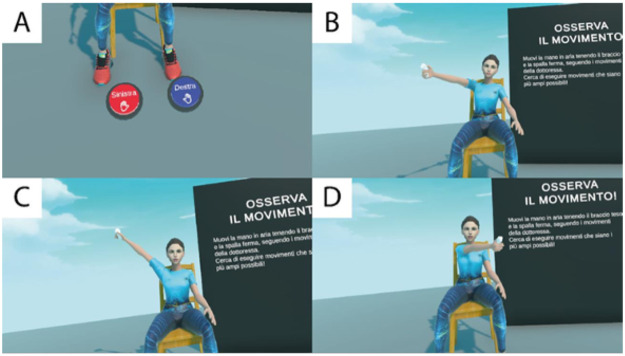
The system calibration phase. In detail, the buttons that allow the selection of the limb to be rehabilitated **(A)**, the combined shoulder movement **(B)**, the abduction movement **(C)**, the frontal expansion movement **(D)**, shown by Dr. FT Bongiorno's avatar.

The calibration phase allows the patient to choose which limb will be used to perform the exercise (upper limb to be rehabilitated) (Figures 1A–D).

**Figure 2:**
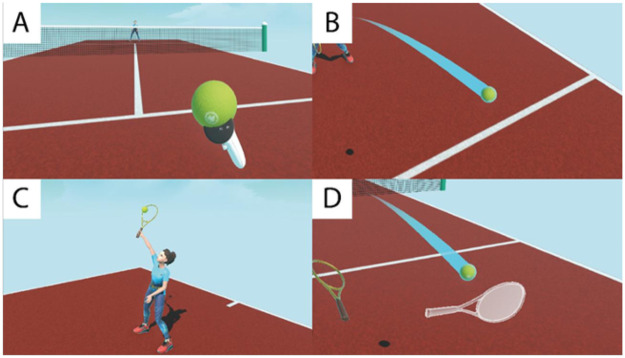
A few moments from the training phase. The ball in the patient's hand ready to serve **(A)**, the colored trail of the ball in flight **(B)**, Dr. FT Bongiorno's serve **(C)**, the guide racket during the tutorial **(D)**

The practice phase involves using the information gleaned from the previous calibration phase to customize the movements required to hit and throw the tennis ball. The patient will be guided in learning the rules of the game, including through a transparent white (ghost) guide racket positioned in the same way as the patient's racket (Figures 2A–D). If the patient misses any shots during the tutorial, her partner will always serve. To avoid the emergence of a competitive spirit and induce stress in the patients, the game does not include scoring, competition, or penalties for errors.

In addition to the software customization achieved during the calibration phase, further adjustments to the virtual system are possible based on the patient's unique clinical features, such as modulating the level of body illusion: the physiotherapist can adjust how much actual shoulder movements differ from virtual shoulder movements. The virtual range of motion can be increased or decreased to create a greater illusion and allow for an increase in range of motion. Based on the subject's range of motion recorded during the calibration phase, the software can exclude movements that exceed the maximum joint range and would cause pain. At the end of the exercise session, the system automatically saves data regarding the patient's performance and difficulties. The report contains the throws, throws, and errors made so that this data can be evaluated by the multidisciplinary team and define the difficulties encountered and the patient's motor performance. At the end of the software testing, each subject was administered 4 validated questionnaires, which investigated the usability of the software, the “User Experience” and the comfort in using the system:

Simulator Sickness Questionnaire (SSQ): A questionnaire that investigates the discomfort and discomfort generated by using Virtual Reality devices (25).
System Usability Scale (SUS): A questionnaire that evaluates the perceived usability of the Virtual Reality system (26).
User Experience Questionnaire (UEQ): A questionnaire that investigates the user's “User Experience” when using the Virtual Reality system (27).
Comfort Rating Scale (CRS): A questionnaire that evaluates the perceived comfort and discomfort when using wearable devices (28).

Furthermore, each subject was asked a question about the validity of the preset speed at which the game is played. The Simulator Sickness Questionnaire (SSQ) is a questionnaire that investigates the occurrence of side effects, discomfort, or discomfort caused by using a Virtual Reality headset. The questionnaire covers a total of 16 symptoms, and subjects can freely add additional symptoms experienced by using the “Other” item at the end of the questionnaire. Some symptoms analyzed by the questionnaire include general malaise, headache, eye pain, or blurred vision. For each item, four levels are identified, in ascending order, corresponding to the intensity of the symptom experienced:

- None: The symptom is not present
- Mild: Slight presence of the symptom
- Moderate: Moderate presence of the symptom
- Severe: Severe presence of the symptom

The questionnaire can be administered before and after using the headset; however, administering the questionnaire before using the VR software can alter the quality of the user's experience by making them aware of the possible onset of general discomfort and, consequently, creating bias in the actual assessment of their health status after using VR. For this reason, in the following study, the SSQ questionnaire was administered exclusively after using VR devices. The System Usability Scale (SUS) is a questionnaire that investigates the usability perceived by users of the virtual system. This 10-item questionnaire, 5 of which are positively weighted and 5 negatively weighted, allows for the assessment of the software's applicability by exploring various areas such as its complexity, the support required for its use, the training required for its use, and the integration of the system's various features. Agreement with each item is expressed through a score ranging from 1 to 5 on a Likert scale, representing “strongly disagree” and “strongly agree,” respectively. The SUS is a clear, short scale to administer and is suitable for evaluating a virtual system. The User Experience Questionnaire (UEQ) is a questionnaire that allows for a quick and immediate assessment of the quality of the user's experience with the Virtual Reality system. The “UEQ” questionnaire allows us to evaluate pragmatic and hedonic aspects: it analyzes characteristics related to the virtual system's usability (efficiency, insight, and reliability), but also to the user's subjective experience, exploring its originality and stimulation. The questionnaire contains 26 items in total, grouped into 6 macro-sections:

- The aesthetic beauty of the software: Do users like the software?
- The clarity of the system: Is it easy to become familiar with the software?
- The efficiency of the software: Are users able to resolve requests without excessive effort?
- The reliability of the software: Do users feel in control of the interaction?
- The stimulation of the software: Is the software stimulating and exciting?
- Originality: Is the software original and creative?

For each of these macro-sections, the questionnaire reserves several of the 26 total items. Each item represents a characteristic of the software and can be described by a pair of contrasting adjectives (e.g., annoying-pleasant, creative-unimaginative, etc.). The user's judgment is based on the choice of a score ranging from 1 to 7: a score of 1 represents the first adjective on the questionnaire (in the example given, 1 corresponds to annoying), while a score of 7 represents the second adjective of the pair (7 corresponds to pleasant). The Comfort Rating Scale (CRS) is a questionnaire developed to investigate the perception of comfort and discomfort during the use of wearable devices. The CRS is a quick and easy questionnaire to administer to evaluate the comfort of wearable virtual systems. The CRS can be used not only to describe the sensation of comfort or discomfort experienced by users, but also to highlight aspects of the device that need improvement and to compare the comfort levels of different wearable systems. The CRS is made up of six dimensions covering the various characteristics that describe the comfort of the wearable system: emotion, attachment, damage, perceived change, movement, and anxiety. To complete the questionnaire, the subject identifies the level of agreement they share with the corresponding item and assigns it a score from 0 to 20. Each of these items has a score from 0 to 20, from left to right: a score of 0 corresponds to a “low” score, while 20 corresponds to a “high” score. Since it was not possible to retrieve the validated version of the CRS questionnaire online, it was decided to independently develop the Comfort Rating Scale following the instructions provided in the respective article. Instructions for completing the questionnaire are provided in Italian and English. Each questionnaire was administered and completed immediately after the software trial and before any discussion or conversation regarding the virtual experience between the examiner and the individual subject took place (25–28). Since the software speed is adjustable, at the end of the trial each participant was asked a question regarding the appropriateness of the preset game speed. The question asked was: “In your opinion, should the game be faster?”, with the option of inserting text fields to complete the response. The text fields inserted in the individual open-ended responses were processed through a qualitative analysis, dividing these responses into thematic categories and allowing for their optimal description and representation (29). The results of the collected questionnaires were then processed (using Microsoft Excel software) and represented as the mean and standard deviation for each item analyzed. The results obtained from each questionnaire were grouped into tables representing the mean, standard deviation, and maximum and minimum value for each item, while the average score for each item in each questionnaire was represented graphically. To analyze the speed results, a qualitative analysis was developed by dividing the most frequently occurring words in the text field available to the subjects into thematic categories. The graphical representation was performed using vertical histograms for the analysis of each questionnaire.

## 3 Results

**Figure 3:**
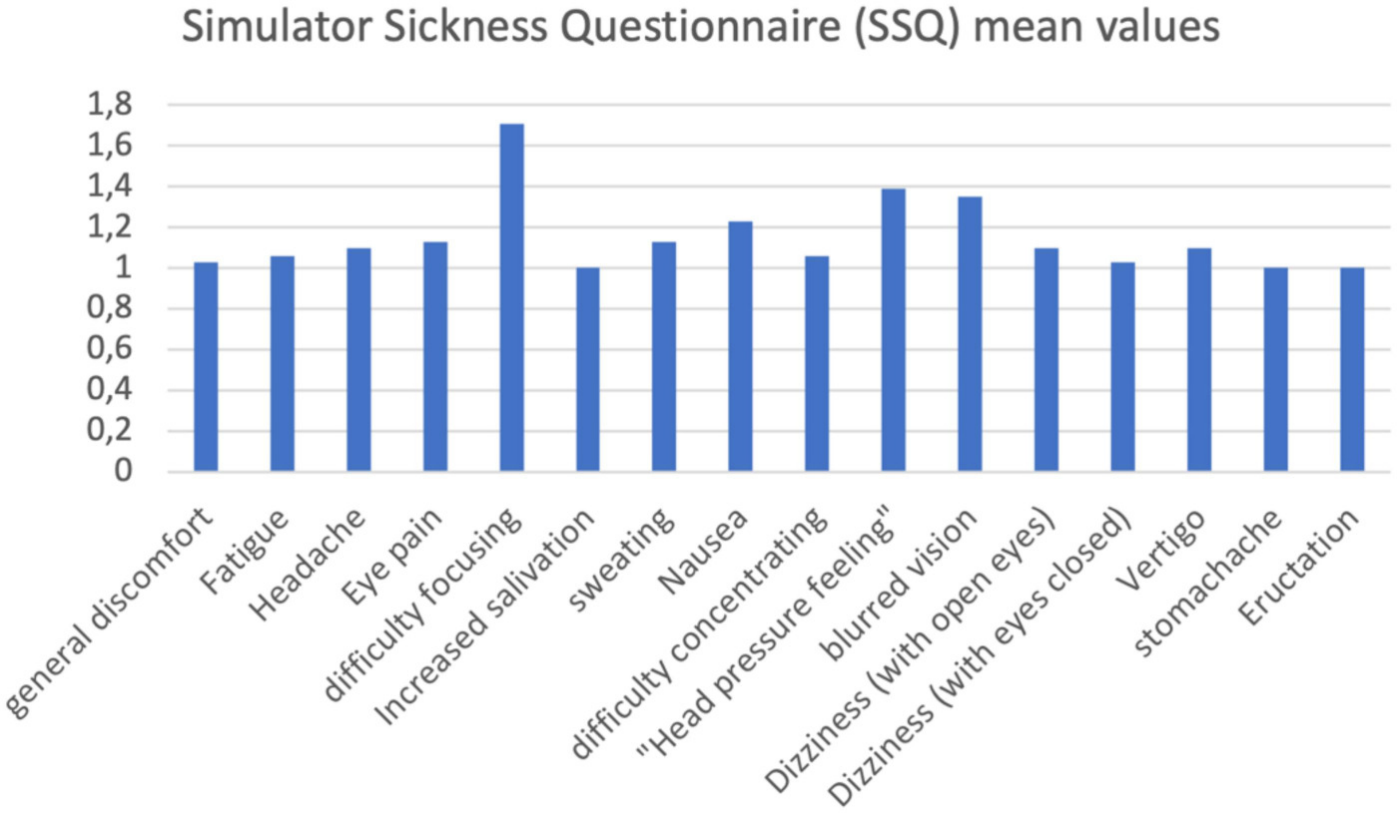
Graphical representation of the mean values for each SSQ item. The items are shown on the *x*-axis, while the *y*-axis shows the mean score for each item.

The results obtained from each questionnaire were grouped into tables representing the mean and standard derivation, while the average score for each item in each questionnaire was represented graphically. To analyze the speed results, a qualitative analysis was developed by dividing the most frequently occurring words in the text field available to the subjects into thematic categories. The graphical representation was performed using vertical histograms for the analysis of each questionnaire. Simulator Sickness Questionnaire Results: Participants responded to 16 items, rating symptoms such as nausea, headache, dizziness, and visual difficulties on a scale from 1 (no symptoms) to 4 (severe symptoms). These results were reported in a table showing the mean, standard deviations, and number of respondents (Table 1). Furthermore, the mean values for each item are shown in a vertical histogram (Figure 3).

**Table 1:** SSQ results for symptoms caused using virtual devices. Mean, standard deviation, minimum, and maximum values are shown for each item.

Item	Total	Mean	Standard deviation
general discomfort	31	1,03	0,18
Fatigue	31	1,06	0,25
Headache	31	1,1	0,3
Eye pain	31	1,13	0,34
difficulty focusing	31	1,71	0,97
Increased salivation	31	1	0
sweating	31	1,13	0,34
Nausea	31	1,23	0,62
difficulty concentrating	31	1,06	0,25
"Head pressure feeling"	31	1,39	0,56
blurred vision	31	1,35	0,66
Dizziness (with open eyes)	31	1,1	0,3
Dizziness (with eyes closed)	31	1,03	0,18
Vertigo	31	1,1	0,3
stomachache	31	1	0
Eructation	31	1	0

### 3.1 System usability scale results

**Figure 4:**
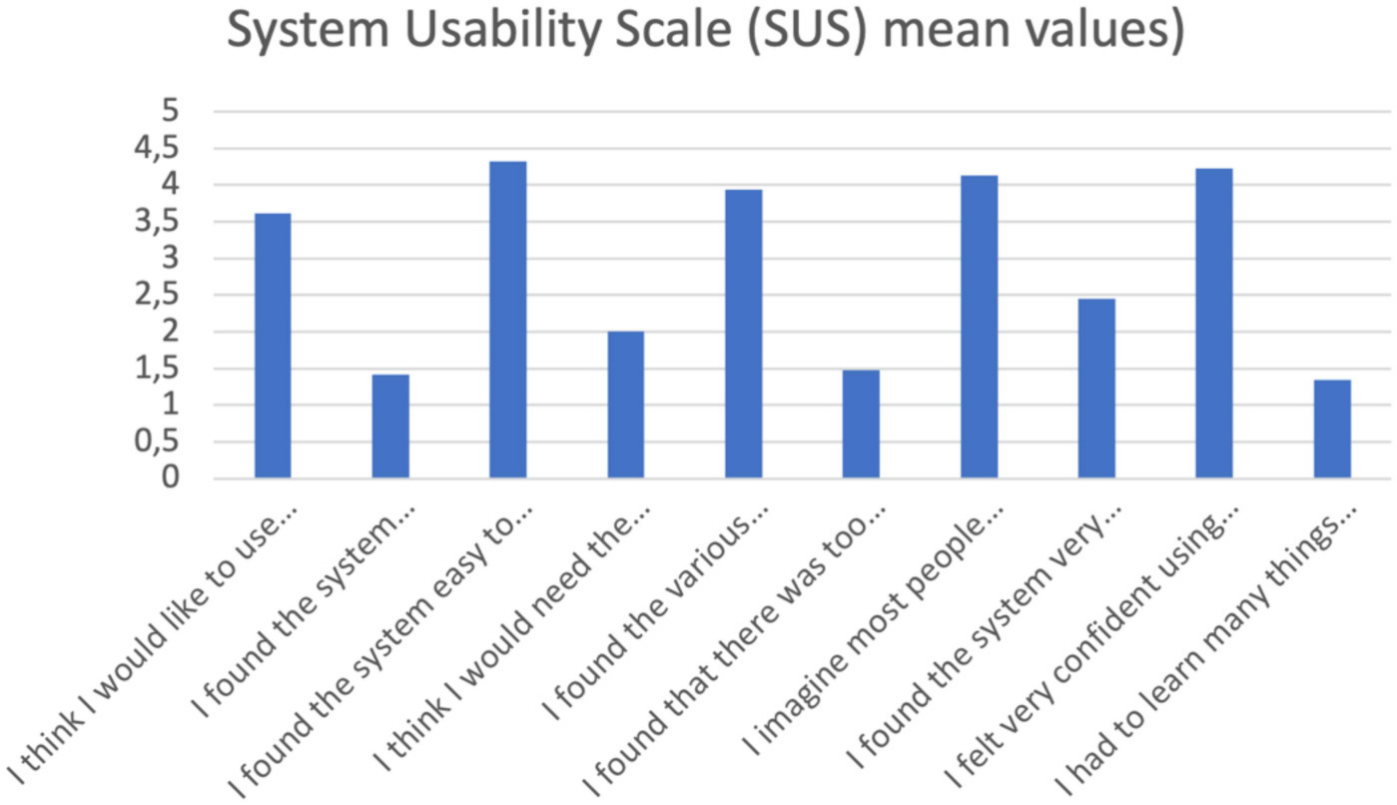
Graphical representation of the mean values for each SUS item. The questionnaire items are shown on the *x*-axis, while the *y*-axis shows the mean score for each item.

Participants responded to 10 statements on a Likert scale ranging from 1 (strongly disagree) to 5 (strongly agree). These results were reported in a table showing the mean, standard deviation, maximum value, number of subjects, and type of each item (Table 2). Additionally, the mean values for each item are shown in a vertical histogram (Figure 4).

**Table 2:** The results obtained from the SUS regarding the applicability of the virtual device by users. Mean, standard deviation, maximum value, minimum value, and item type are shown.

Description	Total	Mean	Standard deviation	Type of item
I think I would like to use this system frequently	31	3,61	1,02	Positiv
I found the system unnecessarily complex.	31	1,42	0,72	Negativ
I found the system easy to use.	31	4,32	1,08	Positiv
I think I would need the support of an experienced person to be able to use this system.	31	2	1,1	Negativ
I found the various functions in the system to be well integrated.	31	3,94	0,85	Positiv
I found that there was too much inconsistency in the system.	31	1,48	0,63	Negativ
I imagine most people would learn to use this system very quickly.	31	4,13	1,06	Positiv
I found the system very cumbersome to use.	31	2,45	1,34	Negativ
I felt very confident using this system.	31	4,23	0,92	Positiv
I had to learn many things before I could use the system.	31	1,35	0,55	Negativ

### 3.2 User experience questionnaire results

**Figure 5:**
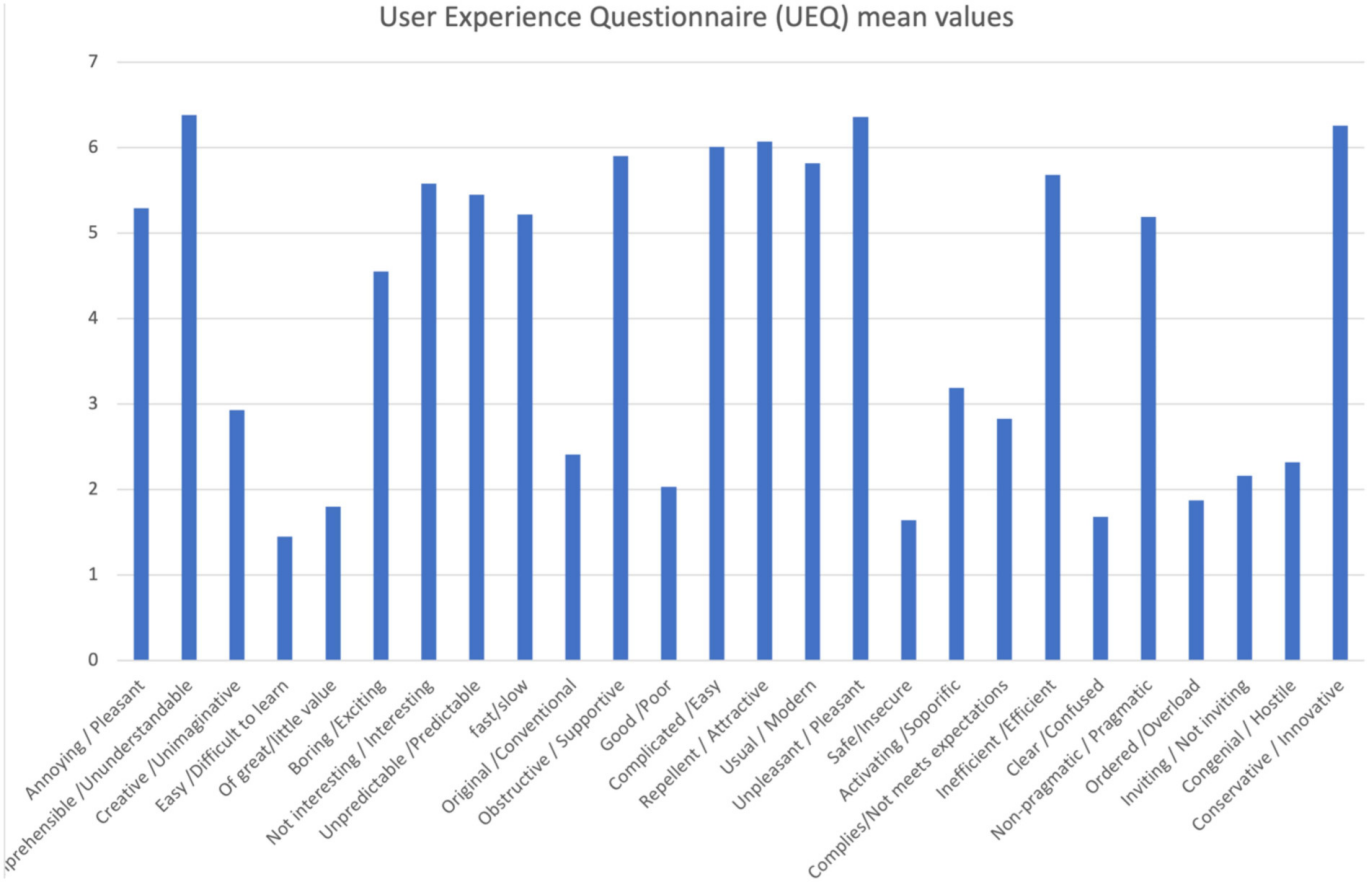
Graphical representation of the mean values of each UEQ item. The questionnaire items are shown on the *x*-axis, while the *y*-axis shows the mean score obtained for each item.

Participants described the quality of their experience with the VR software using 26 items organized into pairs of opposing adjectives (e.g., “pleasant” vs. “annoying”), rated on a scale of 1–7. These results were reported in a table showing: mean, standard deviation, maximum value, and minimum value (Table 3). The mean values for each item are represented in a vertical histogram (Figure 5).

**Table 3:** Data relating to the quality of experience of UEQ participants. The mean, standard deviation, maximum, and minimum values are shown.

Item	Descrizione	Totale	Media	Deviazione standard
Item 1	Annoying/Pleasant	31	5,29	1,51
Item 2	Incomprehensible/Ununderstandable	31	6,38	0,76
Item 3	Creative/Unimaginative	31	2,93	1,48
Item 4	Easy/Difficult to learn	31	1,45	0,51
Item 5	Of great/little value	31	1,8	1,26
Item 6	Boring/Exciting	31	4,55	1,57
Item 7	Not interesting/Interesting	31	5,58	1,41
Item 8	Unpredictable/Predictable	31	5,45	1,15
Item 9	fast/slow	31	5,22	1,33
Item 10	Original/Conventional	31	2,41	1,23
Item 11	Obstructive/Supportive	31	5,9	0,91
Item 12	Good/Poor	31	2,03	1,11
Item 13	Complicated/Easy	31	6,01	1,11
Item 14	Repellent/Attractive	31	6,07	0,87
Item 15	Usual/Modern	31	5,82	0,87
Item 16	Unpleasant/Pleasant	31	6,36	0,81
Item 17	Safe/Insecure	31	1,64	0,8
Item 18	Activating/Soporific	31	3,19	1,58
Item 19	Complies/Not meets expectations	31	2,83	1,19
Item 20	Inefficient/Efficient	31	5,68	1,08
Item 21	Clear/Confused	31	1,68	0,98
Item 22	Non-pragmatic/Pragmatic	31	5,19	1,38
Item 23	Ordered/Overload	31	1,87	0,99
Item 24	Inviting/Not inviting	31	2,16	1,44
Item 25	Congenial/Hostile	31	2,32	1,14
Item 26	Conservative/Innovative	31	6,26	0,77

### 3.3 Comfort rating scale results

**Figure 6:**
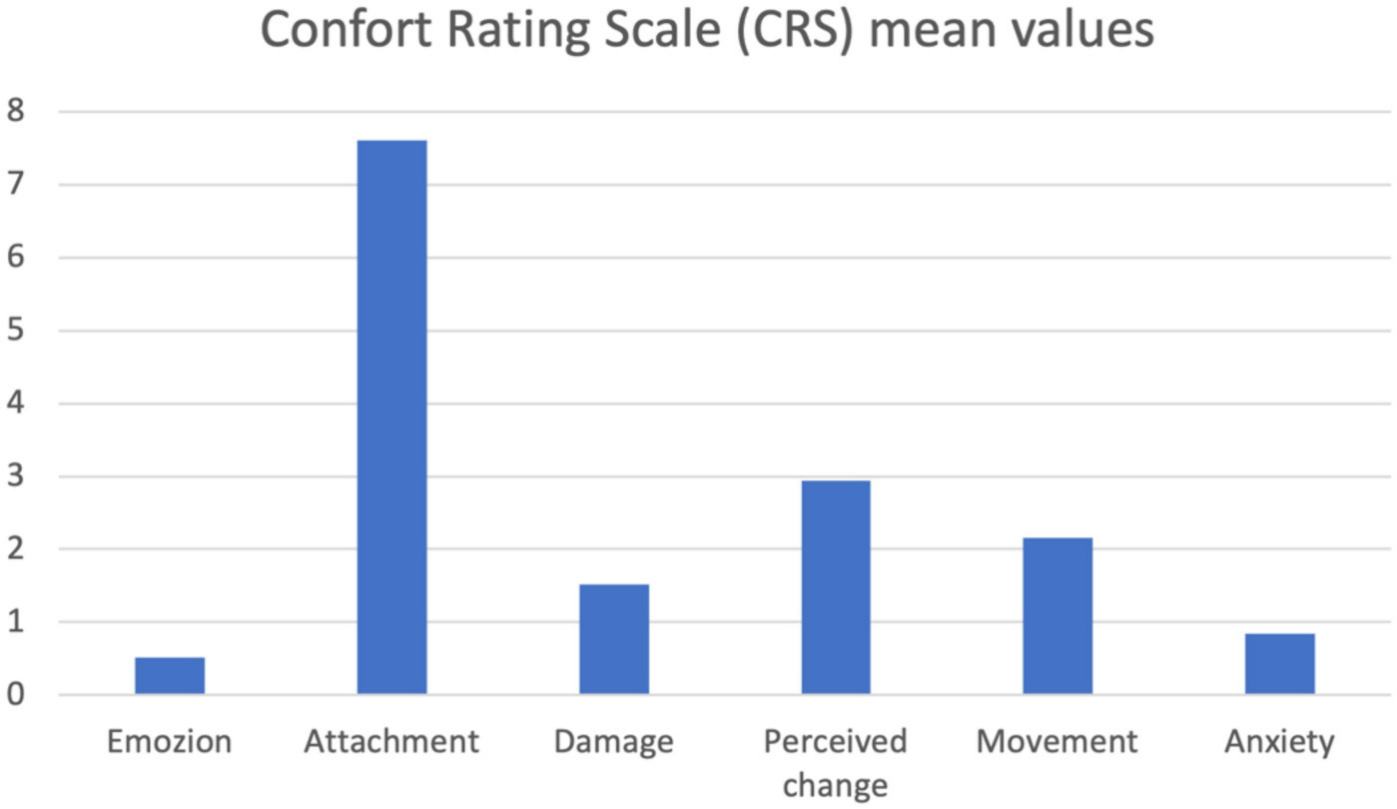
Graphical representation of the mean values for each CRS item. Each item is shown on the *x*-axis, while the *y*-axis shows the mean score obtained for each item.

Participants rated their comfort and discomfort after testing wearable virtual reality devices using a score from 0 to 20. These results were grouped into a table, which represents: mean, standard deviation, maximum value, and minimum value (Table 4). A vertical histogram was also created depicting the mean values for each CRS item (Figure 6).

**Table 4:** Results obtained by the CRS regarding the sensation of discomfort and comfort regarding wearable virtual reality devices. The mean, standard deviation, minimum value, and maximum value are reported.

Item	Total	Mean	Standard deviation
Emozion	31	0,52	0,85
Attachment	31	7,61	6,43
Damage	31	1,52	2,94
Perceived change	31	2,94	4,23
Movement	31	2,16	3,37
Anxiety	31	0,84	1,49

### 3.4 Speed results

**Figure 7:**
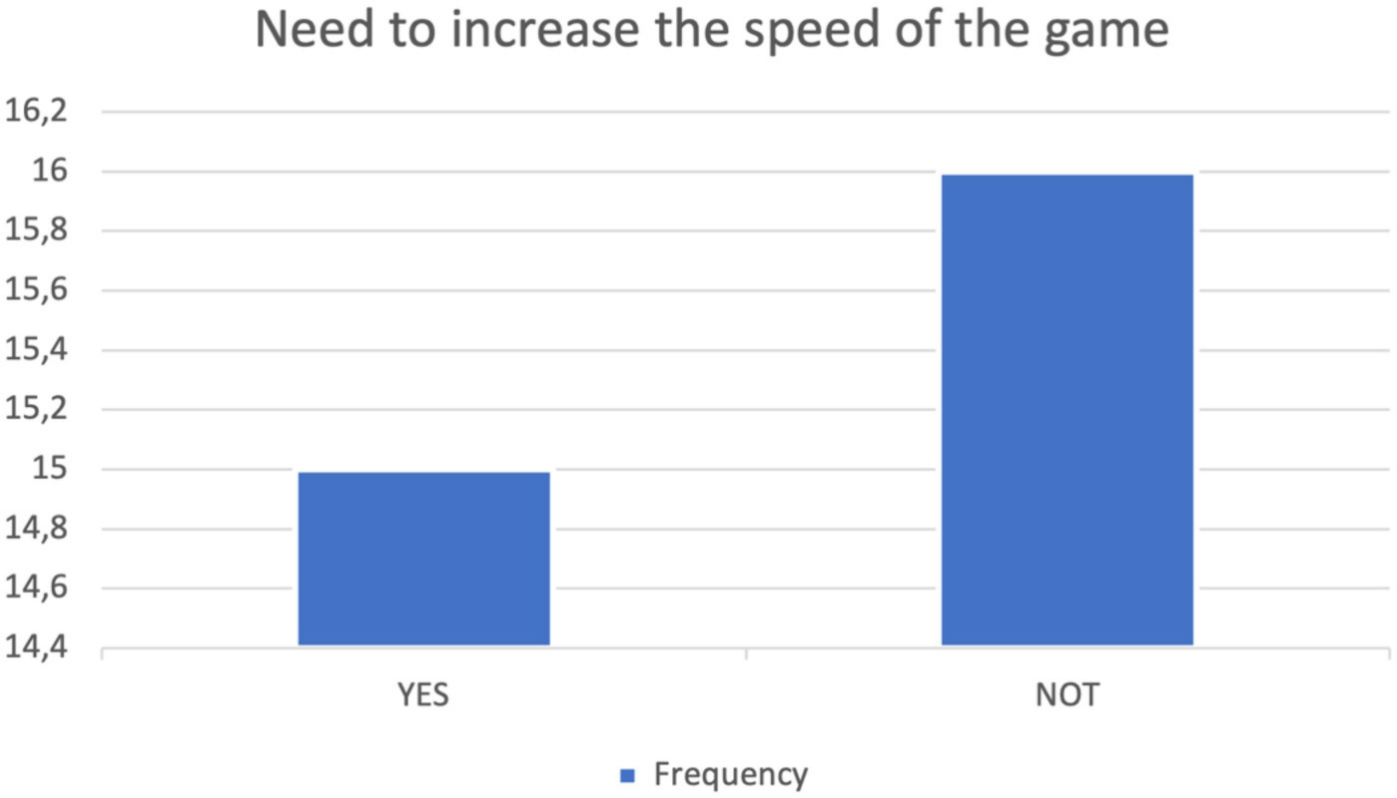
Graphical representation of the distribution of responses regarding the software's preset speed. The *x*-axis shows the question posed to participants, and the *y*-axis shows the frequency of responses obtained.

The results for the preset speed were grouped into a table representing the responses obtained and their respective frequencies (Table 5) and into a vertical histogram representing the distribution of these responses (Figure 7). The statistical analysis for the development of the results yielded 15 “Yes” responses and 16 “No” responses.

**Table 5:** Results obtained from the question about changing the software's preset speeds. The participants' responses and their corresponding frequency are shown.

Answer	Frequency
YES	15
NOT	16

Overall, 31 responses were obtained regarding changing the preset speed, 15 responses for “Yes” and 16 responses for “No”.

## 4 Discussion

The results obtained from the Simulator Sickness Questionnaire show very low mean values ​​for all items, concentrated around 1.0, indicating a minimal level of simulator sickness, or discomfort generated by the virtual device. No study participants reported gastrointestinal symptoms such as burping or stomachache, nor increased salivation. Very low mean values, ranging from 1.03 to 1.71, were recorded for all other mild symptoms, and for each questionnaire item, more than half of the participants gave the corresponding symptom the minimum score. Despite the very low mean values, the three symptoms with the highest mean values ​​were difficulty focusing, a feeling of pressure in the head, and blurry vision. The onset of symptoms generated using virtual devices, even if mild, is frequent, in some cases inevitable, and is constantly studied by researchers; There are various causes attributable to their appearance: from the characteristics of the software itself, its design, but also from individual factors of the single user (10, 23, 30). The visual stimulation provided by the virtual world clashes with the absence of movement stimuli coming from the vestibular system, which generates the onset of symptoms such as nausea and general malaise, symptoms reported in mild intensity in the study conducted. Therefore, the contrast between the contrasting information coming from the two different sensory organs is considered consistent with the symptoms reported by the participants in the study. The correct use of the headset and its optimal positioning on the user's head and face plays a key role in limiting the onset of symptoms after the virtual experience. In fact, an inappropriate positioning of the headset on the head, wearing it excessively tight or loosely or not bringing it well adhered to the face, can lead to the onset of visual symptoms, such as difficulty focusing and blurred vision (10). The sensation of pressure in the head reported by some study participants may be caused by the headset not fitting properly on the head and face. The symptoms reported by healthy study participants following use of the VR device were characterized by very low mean scores and the absence of significant symptoms, indicating the excellent tolerability of the headset and its features (calibration, design, latency period, and software flickering). Analysis of the System Usability Scale results shows that positive items tend to receive moderate scores, indicating a positive perception of the system's usability, while negative items received relatively low mean scores, indicating that the software's usability was not complex or cumbersome. Study participants found the software easy to use and learn, would use it frequently, and felt confident in its use. These results suggest that the features of the proposed software make it suitable for potential future clinical use as a rehabilitation tool for women undergoing breast cancer surgery. The immersion, interaction and imagination that the software offers were positively received by the study participants, giving the virtual system a high level of usability; not only these characteristics, but also the “gamification” of the software and the proposal of virtual gaming with the avatar can determine a greater familiarity in its use, guaranteeing a possible greater adherence to the rehabilitation treatment by the patients in future clinical practice (5, 10, 12). According to other authors (10), assuming a sitting position while using Virtual Reality software and optimal integration of the software's functions can reduce the possibility of developing discomfort and collateral symptoms following the use of virtual devices and increase the user's perceived sense of safety. Although the subjects participating in the study were healthy and found the software safe to use, this result appears very promising for the future clinical use of the device on cancer patients. The ability to distract from painful symptoms that characterizes Virtual Reality is lost in conditions in which it is deemed harmful and dangerous by the user (11), therefore greater safety in using the software implies the possibility of obtaining greater benefits from the use of Virtual Reality. The results obtained from the SUS analysis indicate good tolerability of the software proposed to healthy subjects; the negative items present relatively low average scores, contrary to the positive items which record moderate average values. This suggests optimal usability of the software, making it suitable and appropriate for future clinical use. The results of the User Experience Questionnaire show that the perception of the user experience is generally very positive, with average scores for individual items indicating a very positive quality of experience. The graphical and tabular representation (Figure 5, Table 3) shows that the items relating to simplicity, comprehensibility, and modernity achieve very high average scores, indicating a good reception of the system in terms of ease of use and software innovation. Consistent with the results obtained from the SUS analysis, the item relating to the security or otherwise of the software demonstrates that study participants reported a sense of security in using the proposed software, an encouraging result regarding its future clinical application in oncology. The items relating to the software's aesthetics and clarity recorded relatively high average scores, in line with the minimal symptoms reported by subjects following use of the virtual device and the high usability observed from the SUS analysis. From the analysis of the results, no critical issues emerge, except those relating to the predictability and slowness of the software. The two items respectively have an average score of 5.45 and 5.22, a sign that most of the subjects participating in the study found the software relatively slow and predictable, characteristics that can reduce immersion and excitement for the virtual experience and increase the probability of developing physical discomfort (10). Regarding the preset speed of the software, it is important to specify how the software we have developed finds its first application in the following study: therefore, an average speed was established, and a specific question was developed to be submitted to the sample of professionals and future physiotherapists to evaluate its possible modification. Furthermore, regarding the predictability of the software, it is highlighted that the software developed does not have a recreational purpose, but rather a rehabilitative one: for this reason, it must be as standardized and homogeneous as possible among the users, and its use must be carefully monitored by the medical and rehabilitation team (7, 8). The speed with which the game is developed must also guarantee the safety of the patient, the quality of the movement performed and an appropriate muscular recruitment of the components involved; an excessive dynamism of the game, as occurs in video games, can determine poorly controlled movements and high intensity of the forces acting on the shoulder, limiting and minimizing the real benefits of the virtual experience as well as aggravating the painful symptoms and the clinical condition of the patient (29). The results obtained from the CRS analysis show that the levels of Anxiety and Emotion are almost zero, indicating excellent emotional compliance with the system. The highest average score is recorded for the Attachment item, while the other dimensions—Harm, Movement, Perceived Change—show low or moderate values, suggesting very good overall tolerability of the headset by the students. Consistent with the results obtained from the analysis of the SUS and UEQ questionnaires, the dimensions relating to software safety—Anxiety and Harm—show very low average scores, indicating that the use of the software did not generate a sense of danger or risk in the participants. The dimensions—Attachment and Perceived Change—recorded average values ​​of 7.61 and 2.94, respectively, and represent the two items with the highest average scores. These average values ​​and the relationship between the two dimensions can be attributed to the inadequate positioning of the visor on the user's head and face, increasing the perception of the device on the user's body and altering the quality of the virtual experience, with the consequent risk of developing general malaise (10, 29). The CRS results suggest that the participants positively welcomed the comfort of the software, finding high levels of safety and security during use, thus giving it a high tolerability. As regards the analysis of the results relating to the pre-set speed of the software, the participants took positions that were in clear contrast with each other, indicating that the issue linked to the speed of the software is not obvious and of little value. The frequency with which the participants in the study took the two contrasting positions of “Yes” and “No” when asked for a possible modification of the speed at which the game develops is almost the same, 15 and 16 respectively. These results indicate that, although the overall opinion is divided, a clear need emerges to carefully evaluate the possible modification of the speed of the game: in fact, as previously described, an excessively high speed could determine poorly controlled movements of the shoulder, altering the patient's safety and generating pain (31), at the same time, an excessively low speed would make the software too predictable, impacting on the patients' adherence and motivation to the rehabilitation treatment. It should be noted that the sample in question, namely physiotherapist students, is a convenience sample and cannot be representative of the software's speed. Indeed, the opinion on the game's speed among healthy individuals with no professional experience differs from the opinion of experienced physiotherapists or a sample of individuals with shoulder problems. This element can be considered fundamental for the development of a future study on the use and evaluation of the software in a sample consisting exclusively of physiotherapists with expertise in the oncology field or in a population more closely related to the target population under study. An analysis of the comments reveals that several proposals have been made regarding the software's speed adjustment: some participants propose adjusting the speed based on a specific adaptation of the software to each individual user, while others propose dividing the game into levels. It is essential to highlight how the inclusion of different levels in the game makes the software less homogeneous and standardised. Furthermore, although the use of the software is designed as an autonomous and home-based rehabilitation tool, the rehabilitation process of the woman who has undergone breast surgery will be carefully monitored by the multidisciplinary team, which, in relation to the individual clinical condition and motor performance, will be able to personalise the characteristics of the software (26). The proposal to divide the software into levels appears interesting; therefore, it is possible to evaluate the possibility of ensuring progression in the game in relation to the patient's skills and the speed of the software: the greater the progression through the levels, the faster the game will unfold. Five subjects reported that the game was excessively static, a consideration already noted in the UEQ results. As this was the first time testing the software on a healthy sample, a preset game speed was established to ensure the testing was completely safe for both users and future patients. The remaining subjects believed that the software speed was already adequate for the rehabilitation needs and the type of cancer patients. The results obtained suggest the need to design the Virtual Reality system with parameters that can be adapted or modified, by both the patient and the therapist, in relation to the patient's clinical condition and motor skills. The virtual experience with our software achieved promising and encouraging results for its future use. The study participants found the software to be very easy to use, with a very high level of comfort and low discomfort. Even if it lasted for a limited period, the virtual experience was considered remarkably positive, pleasant and exciting, considering the software to be appropriately integrated, clear and innovative, in line with the quality of patient experience in numerous studies (4, 5, 12, 26, 27, 31). Another interesting topic is given by artificial intelligence in this field both in the general health issue and in the psychological field. The software we developed certainly lends itself to AI development, for example in creating a game-based rehabilitation program that becomes increasingly challenging for the patient as time goes by. Modern machine learning systems can easily develop personalized rehabilitation programs with this game based on the number of errors made by the user and the difficulty/distance from the arm of each “shot” in the last finished training session. However, all of this requires a multidisciplinary development and implementation team, so that innovation is proposed and not imposed on healthcare professionals and patients (32). Among healthcare professionals, an important category of professionals to consider is that of psychologists, who have demonstrated, even in recent experiences (33), that they are ready for this digital transition. Recent experiences of telemedicine in breast oncology (34) report a greater effectiveness of rehabilitation treatment, in terms of quality of life and functionality of the upper limb, in the hospital setting, which may depend on direct supervision of the physiotherapist, greater motivation and adherence, of the patient, continuous personalization of exercises, immediate correction of errors, human intercourse and psychological support. The paper nevertheless reports how telerehabilitation can still be effective, acceptable, affordable and can improve mobility, pain, strength and fatigue. Furthermore, telerehabilitation reduces barriers such as geographical distance, costs, lack of time, and transportation difficulties, proving useful for patients living in peripheral areas, with family responsibilities, and with mobility or work difficulties. There remain some potential intrinsic limitations in this path, such as lack of manual correction in certain situations, technical difficulties (technology, connection), less emotional involvement in certain cases, less motivation, and difficulty adapting exercises in real time. These are certainly limitations that must be considered, but the implementation of AI, the involvement of more professionals, and the ever-increasing degree of digital literacy among the general population will only facilitate this virtuous path.

## Data Availability

The raw data supporting the conclusions of this article will be made available by the authors, without undue reservation.
